# The Prevalence of Pathogenic or Likely Pathogenic Germline Variants in a Nationwide Cohort of Young Colorectal Cancer Patients Using a Panel of 18 Genes Associated with Colorectal Cancer

**DOI:** 10.3390/cancers13205094

**Published:** 2021-10-12

**Authors:** Erik Frostberg, Annabeth Høgh Petersen, Anders Bojesen, Hans Bjarke Rahr, Jan Lindebjerg, Karina Rønlund

**Affiliations:** 1 The Department of Surgery, Colorectal Cancer Center South, Vejle Hospital, University Hospital of Southern Denmark, 7100 Vejle, Denmark; hans.rahr@rsyd.dk; 2 The Danish HNPCC Register, Clinical Research Centre, Copenhagen University Hospital, 2650 Hvidovre, Denmark; 3 The Department of Regional Health Research, Faculty of Health Sciences, University of Southern Denmark, 5000 Odense, Denmark; 4 The Department of Clinical Genetics, Vejle Hospital, University Hospital of Southern Denmark, 7100 Vejle, Denmark; Annabeth.Hogh.Petersen@rsyd.dk (A.H.P.); Karina.Roenlund2@rsyd.dk (K.R.); 5 The Department of Clinical Genetics, Skejby University Hospital, 8200 Aarhus, Denmark; ANDBOJ@rm.dk; 6 The Department of Pathology, Colorectal Cancer Center South, Vejle Hospital, University Hospital of Southern Denmark, 7100 Vejle, Denmark; jan.lindebjerg@rsyd.dk

**Keywords:** colorectal cancer, germline mutation, germline variants, Lynch syndrome, nationwide, young

## Abstract

**Simple Summary:**

This study reveals that pathogenic or likely pathogenic germline variants are detected in one fourth of all young patients with colorectal cancer in a nationwide cohort. Immunohistochemistry staining for mismatch repair deficiency is an easy way to detect Lynch syndrome in the young colorectal cancer patient.

**Abstract:**

Introduction: The prevalence of pathogenic or likely pathogenic germline variants (PGV) in colorectal cancer (CRC) in young patients is seen in approximately one in five patients, with the majority of cases having gene variants associated with Lynch syndrome (LS). The primary aim was to describe the prevalence of 18 genes, all associated with hereditary polyposis and CRC, in a nationwide population of young CRC (yCRC) patients, and outline disease characteristics in patients with or without germline variants. Methods: We screened 98 patients aged 18–40 with CRC diagnosed in 2010–2013 for variants in *MSH2*, *MSH6*, *MLH1*, *PMS2*, *EPCAM*, *APC*, *MUTYH*, *SMAD4*, *BMPR1A*, *STK11*, *PTEN*, *POLE*, *POLD1*, *NTHL1*, *AXIN2*, *MSH3*, *GREM1* and *RNF43* using Next Generation Sequencing. Comparisons between patients’ characteristics in patients with PGV, and patients without germline variants (NPGV) were analyzed. Results: PGV were detected in twenty-four patients (24.5%), and twenty-one patients (21.1%) had variants in the mismatch repair (MMR) genes associated with LS. Variants in the *APC* and *MUTYH* genes were detected in 1% and 4%, respectively. Patients with NPGV had more advanced disease with adverse histopathological features. Conclusion: PGV was detected in one in four yCRC patients, and one in five yCRC patients had disease causing variants in the mismatch repair genes associated with LS.

## 1. Introduction

Colorectal cancer (CRC) is one of the most common cancer forms worldwide, and in Denmark approximately 5000 patients are diagnosed each year, with an median age at diagnosis of 71 years [1]. A small fraction of the CRC population consists of young patients (yCRC) diagnosed at the age of 40 years or younger; however, the incidence of yCRC is reportedly increasing worldwide, including in Europe [2].

Hereditary cancer syndromes have been reported in approximately one out of five yCRC patients, most of them with Lynch syndrome (LS) [3,4,5,6]. LS is characterized by pathogenic variants in the mismatch repair (MMR) genes, and the reported prevalence in the yCRC population varies between 6 and 22% [3,4,7,8,9,10,11,12,13]. Familial adenomatous polyposis (FAP), *MUTYH*-associated polyposis (MAP), Peutz-Jeghers syndrome (PJS), Juvenile polyposis syndrome (JPS) and Cowden syndrome (CS) are other known hereditary cancer syndromes associated with CRC. The prevalence of FAP in yCRC is reported to be 1–8% [3,4,8,9,12,13], while the prevalence for the other syndromes in the yCRC population is generally considered to be low [3,4,5,13,14,15]. The variation in the reported prevalence may be due to variation in the methods used, differences in age definition of yCRC (some use 50 as the upper age limit) and single-center study design. Likewise, the prevalence of newly discovered novel hereditary colorectal cancer genes [16], is not fully investigated in the yCRC patients. Finally, a positive family history with CRC is reported in 18–39% of sporadic yCRC [8,17,18], and thus a considerable proportion of yCRC patients has no known genetic predisposition for their cancer.

The aim of this study was to investigate the frequency of pathogenic or likely pathogenic germline variants, all associated with CRC, in a national cohort of yCRC patients having bowel resection surgery in the years 2010–2013. Further, the aim was to compare disease characteristics between patients with pathogenic or likely pathogenic germline variants and patients without these variants.

## 2. Results

Tissue samples from 104 patients were available for analysis; however, no normal tissue was found in the specimens from six patients and, therefore, 98 patient samples were subjected to variant screening of 18 CRC related genes using Next Generation Sequencing (NGS). The sequencing of the 98 samples resulted in an average coverage of 146× (55×–276×) across the complete target region (90 genes). We found that 99.3% (98.3–99.7%) of total reads could be aligned to the human reference genome hg19, and 96.5% of the target region had a minimum of 30× coverage (88.7–97.4%). Furthermore, 98.5% of the target region of the 18 genes associated with CRC had a 30× coverage (94.6–99.9%) across the 98 samples. These results are comparable to the validation study of this NGS approach, where 40 blood samples resulted in the following quality statistics: 99.4% of total reads were aligned to hg19, average coverage of 210× (141–311) and 97.3% of the target region had a minimum coverage of 30×.

### 2.1. Patients with Pathogenic or Likely Pathogenic Germline Variants

PGV were found in 24 patients (24.5%), of whom 22 patients had one PGV, one patient had two PGV while one patient had three PGV (Table 1). The median age at diagnosis was 35 years (range: 18–40), and 14 patients were females. One patient had rectal cancer, 20 patients had colon cancer and three patients had synchronous CRC. The majority of the tumors were located in the right colon and were traditional adenocarcinomas, and most of the patients were diagnosed in UICC stage I and II. Deficient MMR (dMMR) was seen in 20 (83%) patients, and 13 (54%) had a first degree relative (FDR) with CRC. Eleven patients (10 with LS, 1 with FAP) had extended resection surgery (colectomy or proctocolectomy), and the others had segmental resection. Among these, nine patients had LS.

PGV were seen in the MMR genes (*MLH1*: twelve patients, *MSH2*: seven patients, *MSH6*: one patient, *PMS2*: one patient), *MUTYH* (three patients with monoallelic, and one patient with biallelic variants), and *APC* gene (one patient) (Table 1). LS was seen in 21 patients (21% of the entire analyzed cohort), and 20 of them had deficient MMR by IHC staining. Patients with *MLH1* PGV had loss of expression in *MLH1* and *PMS2*, and patients with *MSH2* PGV had loss of expression in *MSH2* and *MSH6*. One patient with *MSH2* PGV showed loss of expression in all four MMR genes. The patient with *MSH6* PGV only had loss of expression in *MSH6*, and the patient with *PMS2* PGV had isolated loss of *PMS2* by IHC staining. One patient with LS (*MLH1*, c.298C>T) and the three patients with monoallelic *MUTYH* and *APC* PGV showed normal expression of MMR, and thus had proficient MMR (pMMR) stained with IHC.

### 2.2. Patients with No Reported Pathogenic Germline Variants

The median age was 36 years (range: 22–40) in the non-PGV (NPGV) group. An equal distribution of right-sided, left-sided and rectal tumors were seen, and only one patient had synchronous cancer. The majority of tumors were adenocarcinomas, and most of the patients were diagnosed in UICC stage III (37%) and IV (26%). Three patients had known pre-disposing factors (Crohn’s disease: *n* = 2; Ulcerative colitis: *n* = 1). Ninety percent of the patients had segmental resection surgery.

Ninety-two percent of the NPGV patients had pMMR, and only one fifth of the patients had a FDR with CRC. Six patients had dMMR by IHC staining, and a supplementary data analysis of VUS was performed for these patients (Table 2). One patient (id 2.3) had loss of *MSH2* and *MSH6* expression, and patient 2.2 had isolated loss of expression in *PMS2*. Three patients, 2.1, 2.4 and 2.5, had loss of *MLH1* and *PMS2* expression, and patient 2.6 had isolated *MLH1* loss of expression (*MSH6* and *PMS2* were not tested). Patient 2.1 showed no hypermethylation of *MLH1* promoter, and a VUS, *MLH1* c.1996T > C was detected. The *MLH1* c.1996T > C variant is classified by the International Society for Gastrointestinal Hereditary Tumours (InSiGHT) as a VUS (InSiGHT class 3), and due to the patient’s loss of *MLH1* expression additional functional analysis could be of relevance. Data were not available regarding testing for hypermethylation of the *MLH1* promotor in patient 2.4, 2.5 and 2.6 with loss of *MLH1* expression. However, patient 2.4 was tested for *BRAF* and no mutation was found in *BRAF V600E*, but a VUS in *PMS2* was detected in this patient using NGS. The *PMS2* c.857A > G variant is not reported to or classified by InSiGHT, but the variant has been reported 11 times to ClinVar as a VUS.

### 2.3. Differences between PGV Carriers and Non-Carriers

An equal gender distribution was seen between groups. Significantly more NPGV tumors were located in the left colon or rectum, and more tumors in the NPGV group showed adverse histopathological features such as vascular invasion and tumor budding. Almost 60% had lymph node metastasis in the NPGV group compared to only 30% of patients with PGV, and higher UICC stages were seen in the NPGV group. The proportions of dMMR and FDR with CRC were higher in the PGV group. All patient characteristics for both groups are listed in Table 3.

**Figure 1:**
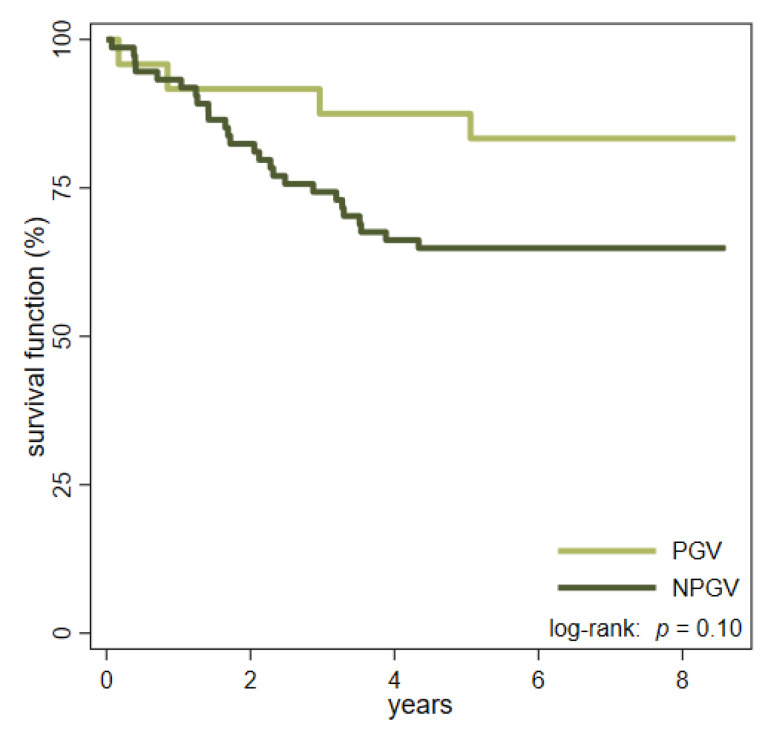
Kaplan-Meier curves estimating the overall survival in patients with pathogenic germline variants (PGV) and no pathogenic germline variants (NPGV).

The median observation time for PGV patients was 80 months (range 2–104 months), and four patients (17%) died in the study period. In the NPGV group, 26 patients (35%) died during a median follow-up time of 72 months (range 0–103 months) (Figure 1). Seventeen of the patients in the NPGV group, and one patient in the PGV group, who died, had disseminated disease at the time of diagnosis.

## 3. Discussion

This study showed that one out of four yCRC patients have a pathogenic or likely pathogenic germline variant, most of them related to LS (one in five patients), and the prevalence of *MUTYH* and *APC* variants was 4% and 1%, respectively. The majority of PGV patients had right-sided tumors, and a positive family history with CRC was more common in PGV patients than in NPGV patients. Patients in the NPGV group were more likely to have adverse histopathological features, although only lymph node metastasis emerged as being statistically significant, and the overall survival seemed to be better in the PGV group. The better survival in PGV patients, and the lower cancer stages in this group, may be due to the fact that one third of the PGV patients were related to known families in the Danish hereditary non-polyposis colorectal cancer (HNPCC) register. Therefore, their cancer may have been found during surveillance colonoscopy, or the patients may have been more aware of symptoms of CRC than NPGV patients with no familiar disposition.

Our data showed a similar prevalence of PGV in yCRC as the recent literature. PGVs were reported in 88 (26%) out of 333 young onset (<50 years old) CRC patients with pMMR or unknown MMR status in a multinational multicenter study by DeRycke et al. [13]. Stoffel et al. [3] found PGV in 79 of 315 (25%) early onset (<50 years old) CRC patients in a single tertiary center cohort. Pearlman et al. [4] reported a lower prevalence and found PGVs in 72 of 450 (16%) early onset CRC (<50 years old) patients having surgical resection in a statewide cohort in the United States. All studies used a multigene panel with CRC associated genes similar to our study, but genes related to other various cancer syndromes not directly related to CRC (e.g., *BRCA1*, *BRCA2*, *ATM*, *ATM/CHEK2*, *PALB2, RECQL5* and *CDKN2A*, etc.) were also included in three studies. Stoffel et al. [3] found that 1% of the sequenced patients had non-traditional CRC germline variants. Pearlman et al. [4] and DeRycke et al. [13] reported a slightly higher prevalence of pathogenic variants in these genes, 3% and 6%, respectively. Our study is not directly comparable with the other studies, since we used a younger population and the variance classification differs in respective study. It is likely that our overall prevalence would be higher if our gene panel also included genes not traditionally associated with CRC. However, to screen for germline variants in genes involved in different cancer pathways not traditionally associated with CRC is controversial, and the interpretation of the results may be difficult. This is a known dilemma in the setting of the genetic counselling, and has been discussed and questioned by others [19,20]. This was one reason why a more exploratory approach was not appropriate for this study. Another reason was the fact that this study included patients diagnosed with CRC at a time when broad routine screening for PGVs were not yet recommended. A recommendation of screening for 18 genes associated with CRC in CRC patients <50 years was first published in national guidelines from the Danish Society of Medical Genetics (DSMG) in 2017 [21]. The main objective was therefore to elucidate the prevalence of PGVs in our historical cohort of young CRC patients based on present-day DMSG guidelines, and our permission from the Regional Scientific Ethical committee was restricted to these particular 18 genes. Our gene panel included 18 genes, but PGVs were only detected in the MMR genes, *APC*, and *MUTYH*. It was not possible to test for the known 40 kb duplication, associated with CRC, located upstream of *GREM1*, since CNV calling has not been validated in libraries obtained from DNA extracted from FFPE samples used in this study. PGV in the other genes associated with CRC and polyposis were not found and must be seen as rarely occurring in the yCRC population. The genes selected in the panel used in this study are described in a recent review [16], and do not include the *TP53* gene related to LiFraumeni syndrome (LFS) which has been seen in six patients (≈1%) in a population-based cohort of 457 yCRC patients (<40 years old) [9]. The phenotype of LFS is characterized by multiple primary cancers in different organs and is not specifically related to CRC and was not recommended in the Danish guidelines on screening for hereditary colorectal cancer syndromes at the time of the analysis for this study [21].

One patient with a pathogenic germline variant in *APC* also had biallelic (compound heterozygous) pathogenic *MUTYH* variants, and three patients (3%) had monoallelic variants in *MUTYH* (one patient also had PGV in MLH1) which is reported in 1–2% of the general population [22], and in 3% in a Spanish multicenter early onset CRC (<50 years old) population [8]. Our finding is interesting, since the cumulative risk of CRC in young monoallelic *MUTYH* carriers is very low and estimated to be 0.0 in 30-year-olds, and 0.2 in 40-year-olds, compared to a risk of 0.01 and 0.07 in the general population at the same age [23]. One patient had a pathogenic variant in *MSH6*. Patients with pathogenic variants in *MSH6* are mostly diagnosed with CRC at older ages [24], but *MSH6* germline variants were described in six out of eleven early onset CRC patients with PGV in the MMR genes in the Spanish cohort mentioned above [8].

Both the Amsterdam criteria and the revised Bethesda guidelines recommend testing for deficiency in the mismatch repair in patients younger than fifty years old [25,26]. Deficient MMR was seen in 20 of 21 patients with LS in our study, while proficient MMR was seen in 68 of the 74 patients in the NPGV group. This corresponds to 95% sensitivity and 92% specificity for MMR testing by IHC. Recent studies have reported poor utilization of MMR deficiency, and data from the National Cancer Database—a population-based database in the United States—showed that only 2174 (50%) of 4381 yCRC patients (<40 years old) were tested for MMR deficiency [27]. Another study reports utilization of MMR staining in only 23% of early onset CRC patients (<50 years old) [28]. This is consistent with a survey among, particularly, gastroenterologists in the US showing inconsistency and uncertainty as to who is responsible for requesting the MMR IHC staining in young adults with CRC [29]. Assessments of the IHC MMR status have been recommended in national guidelines in Denmark since 2005 [30]. Data were not available as to whether the result of the MMR staining was known or considered before surgical treatment decisions, but half of the patients with LS, in this study, had only segmental resection. This could suggest that IHC MMR status, and/or results from MLH1 hypermethylation, was not taken into consideration before surgery. On the other hand, we cannot rule out the possibility that the patient and/or the surgeon settled for less extensive surgery after careful consideration in spite of Danish as well as international recommendations [31,32].

The major strength of this study is that we screened a nationwide cohort of young patients with CRC in an effort to minimize selection bias. In addition, we used the same multigene panel for all patients in one central laboratory unit, avoiding technical biases. The main limitation was the necessity to exclude 55 of 174 (32%) patients in the four year cohort. The main reason for this was the retrospective nature of this study, and in particular since biopsies from non-operated patients and tissue samples from patients having neo-adjuvant treatment were not considered to contain the normal tissue needed for the DNA sequencing. As mentioned, CNV calling in libraries obtained from DNA extracted from FFPE samples has not been validated for the used NGS approach. However, CNV calling was performed but no larger exon duplications or deletions were detected in the analyzed patient samples. Furthermore, the detection of structural variants, such as larger CNVs, inversions, insertions and translocations of genomic DNA segments, has not been validated and is a limitation of this short read NGS approach.

## 4. Materials and Methods

### 4.1. Study Population

For this study, a subpopulation of a national cohort of yCRC patients, all with histologically verified CRC in the age range of 18–40 years old in the years of 2001–2013 and described elsewhere [33], was used. Briefly, 174 patients diagnosed in four consecutive years (1 January 2010–31 December 2013) were of interest and due to pathological assessment and the need for normal-tissue-only patients having resection surgery with no prior radio-chemotherapy were included. Tissue samples from 119 eligible yCRC patients were requested from all pathological departments in Denmark. Specimens from fifteen patients were not available and, in total, biological material from 104 yCRC patients was analyzed.

### 4.2. Clinical and Pathological Data

Demographic, treatment and survival data were available through the national colorectal cancer database [34]. The database holds prospectively collected data for all patients in Denmark with first-time CRC, and due to the unique civil registration number given to all Danish citizens by birth or immigration, linking to other national registers is possible. Data were cross-linked with the HNPCC register, a national register founded in Denmark in 1991 with the aim of identifying families with hereditary CRC and improving their prognoses [35].

Histopathological data on tumor size, histological tumor type, grade of malignancy/degree of differentiation, number and involvement of lymph nodes, and MMR staining with immunohistochemistry (IHC) were collected from the pathological reports and clinical registers. More advanced pathology was investigated using a sample from the invasive front to assess venous and perineural tumor invasion, tumor budding [36], and epithelial-stromal ratio [37]. The assessments were performed by a single gastro-pathologist blinded to the patients’ clinical and demographic data (JLI).

Right-sided tumors were located in the caecum, ascending colon and the transverse colon, left-sided tumors were located in the splenic flexure, descending and sigmoid colon, and rectal cancers were located ≤15 cm from the anal verge. The stage of the disease was classified using the T, N and M stage according to the Union for International Cancer Control (UICC). Deficient MMR (dMMR) was defined as loss of expression of at least one of the tested MMR proteins (by IHC).

### 4.3. Molecular Analysis

Nine sections with a thickness of 15 microns were cut from a paraffin block from each patient containing normal tissue fixed in formalin. Genomic DNA (gDNA) was extracted using the Maxwell^®^16 Instrument (Promega Corporation, Madison, WI, USA) with Maxwell^®^16 FFPE Plus LEV DNA Purification Kit. The quality and quantity of gDNA was evaluated with Pronex DNA Quality Control Assay (Promega Corporation, Madison, WI, USA), a human-specific qPCR assay that detects 75 base pairs (bp), 150 bp and 300 bp using Applied Biosystems™ QuantStudio™ 12K Flex (Applied Biosystems, Thermo Fisher Scientific, Waltham, MA, USA) as analyzing tool. The concentration of gDNA was measured with Qubit^®^ 3.0 Fluorometer (Thermo Fisher Scientific, Waltham, MA, USA). In addition, gDNA was also verified by electrophoresis using Agilent Genomic DNA ScreenTape (Agilent Technologies, Inc., Santa Clara, CA, USA).

The gDNA was fragmented using Covaris ME220 Focused-ultrasonicator™ (Covaris, Inc., Woburn, MA, USA) and afterwards subjected to TruSeq library (Illumina, Inc., San Diego, CA, USA) preparation. Truseq libraries were validated by electrophoresis using Agilent D1000 ScreenTape (Agilent Technologies, Inc., Santa Clara, CA, USA), and the library size distribution was around 200–500 bp. The libraries were pooled, hybridized with custom designed (available upon request to the authors) xGEN Lockdown probes (Integrated DNA Technologies, Inc., Skokie, IL, USA) and captured. The targeted library pools were validated and quantified using Agilent High Sensitivity D1000 ScreenTape (Agilent Technologies, Inc., Santa Clara, CA, USA). The pools were diluted and denatured before a library pool (8 pM) containing 18–20 libraries was subjected to paired-end (2 × 150 bp), dual indexing (2 × 8 bp) Next Generation sequencing on a MiSeq (Illumina, Inc., San Diego, CA, USA).

The selected genes for analysis included genes causing LS (*MSH2*, *MSH6*, *MLH1*, *PMS2*, *EPCAM*), FAP/MAP (*APC*, *MUTYH*), JPS (*SMAD4*, *BMPR1A*), PJS (*STK11*) and CS (*PTEN*), all known genetic causes of hereditary cancer syndromes with a main colorectal component of cancer. Novel hereditary colorectal cancer genes were also analyzed; these genes include *POLE*, *POLD1*, *NTHL1*, *AXIN2* and *MSH3,* which are associated with an adenomatous polyposis or attenuated adenomatous polyposis phenotype. *GREM1* is associated with hereditary mixed polyposis syndrome, and *RNF43* is associated with serrated polyposis. The gene analysis included 18 genes that are recommended to be examined in all patients with colorectal cancer before fifty years of age according to guidelines from the DSMG [21].

For each sequenced sample, the raw fastq files generated from the Illumina MiSeq (Illumina, Inc., San Diego, CA, USA) system were trimmed with TrimGalore (The Babraham Institute, Cambridge, UK) (version 0.4.2), subsequently mapped to the hg19 reference genome using BWA [38] (version 0.7.15) and converted to BAM using Samtools [39] (version 1.3.1). Each sample BAM file was preprocessed with Genome Analysis Toolkit [40,41] (GATK version 3.6; local realignment around indels and base quality scores recalibration) before variant calling. General alignment statistics (e.g., number of aligned reads, size and insert fragments, etc.) were generated with BAMtools [42] (version 2.3.0). Following preprocessing of BAM files, variant calling was performed using GATK HaplotypeCaller. Each call set was annotated using SnpEff [43] (version 4.3). VCFtools [44] (version 0.1.15) was used to generate vcf files for further data analysis using VarSeq (Golden Helix, Inc., Bozeman, MT, USA).

The interpretations of the sequence variants were classified in five categories; pathogenic, likely pathogenic, variant of uncertain significance (VUS), likely benign and benign using the American College of Medical Genetics (ACMG) standards [45]. In this study, only variants classified as pathogenic or likely pathogenic were included in the analysis.

### 4.4. Statistical Methods

Patients were divided into two groups, one group of patients with pathogenic or likely pathogenic germline variants (PGV) and another group without reported variants (NPGV). Comparisons of patient characteristics between the two groups were analyzed with the Fisher’s exact test or the *χ2*–test (chi-squared) depending on expected observation size. The results are presented in tables and text as number of observations as well as proportions. Unknown values, if any, were noted for each variable in tables. Kaplan–Meier estimates and the log-rank test were used to compare overall survival (OS) between groups. OS was time in days from surgery until death; otherwise, patients were right censored at the end of observation time (12 February 2019). All patients had minimum five years of follow-up, and none were lost to follow-up. *p*-values <0.05 were considered statistically significant, and statistical analysis was performed in Stata IC/15.0 (StataCorp, 4905 Lakeway Drive, College Station, TX 77845 USA).

### 4.5. Ethical Considerations

This study was approved by the Danish Colorectal Cancer Group (2013–03, ”CRC hos unge i DK”), the Danish Data Protection Agency (2008-58-0035) and the Regional Scientific Ethical Committee for Southern Denmark (S-20130079). The latter approved the project with an exemption from the rules on obtaining consent from the patients (Sundhedsloven §46. Stk. 1). Furthermore, all patients were screened in the Danish Registry for Use of Tissue, a register holding information about whether patients have declined scientific use of their biological material. None of the patients included in this study were registered at 18th October 2018 when the analysis began. If germline pathogenic or likely pathogenic variants were found in patients who had not previously been diagnosed with a hereditary colorectal cancer syndrome, genetic counseling was offered for these patients and families.

## 5. Conclusions

We found pathogenic germline variants associated with CRC in one out of four young patients with CRC, but the remaining three out of four yCRC patients could not be ascribed to known genetic risk factors. The latter group tended to have more advanced disease and more unfavorable histopathology at diagnosis.

## Figures and Tables

**Table 1:** A list of patient characteristics in 24 young colorectal cancer patients with germline pathogenic variants (PGV). All patients were heterozygous, except patient 1.23 who was compound heterozygous.

Id	Gene	Variant (c.)	Variant (*p*.)	MLH1	MSH2	MSH6	PMS2	FDR	Sex	HIS	TL	UICC
1.1	*MLH1*	c.1276C > T	p.(Gln426Ter)	L	N	N	L	Yes	M	AC	RS	III
1.2	*MLH1*	c.1667 + 2_1667 + 8delinsATTT		L	N	N	L	Yes	F	AC	RS	III
1.3	*MLH1*	c.2041G > A	p.(Ala681Thr)	L	N	N	L	NA	M	AC	RS	II
1.4	*MLH1*	c.2041G > A	p.(Ala681Thr)	L	N	NA	NA	NA	F	AC	RS	III
1.5	*MLH1*	c.298C > T	p.(Arg100Ter)	N	N	N	N	Yes	M	AC	RS	I
1.6	*MLH1*	c.350C > T	p.(Thr117Met)	L	N	N	L	Yes	F	AC	RS	I
1.7	*MLH1*	c.350C > T	p.(Thr117Met)	L	N	N	L	NA	M	AC	SY	II
1.8	*MLH1*	c.350C > T	p.(Thr117Met)	L	N	N	L	No	F	AC	RS	II
1.9	*MLH1*	c.677 + 3A > T		L	N	N	L	NA	F	AC	LS	II
1.10	*MLH1*	c.67delG	p.(Glu23Lysfs*13)	L	N	N	L	Yes	F	AC	RS	I
1.11	*MLH1*	c.76C > T	p.(Gln26Ter)	L	N	N	L	Yes	M	MUC	SY	II
1.12	*MSH2*	c.1165C > T	p.(Arg389Ter)	L	L	L	L	Yes	F	AC	RS	II
1.13	*MSH2*	c.1276 + 1G > T		N	L	L	N	Yes	M	AC	SY	II
1.14	*MSH2*	c.1759 + 2T > G		N	L	L	N	No	M	AC	RE	II
1.15	*MSH2*	c.1786_1788delAAT	p.(Asn596del)	N	L	L	N	No	F	AC	LS	II
1.16	*MSH2*	c.892C > T	p.(Gln298Ter)	N	L	L	N	Yes	M	SRC	RS	I
1.17	*MSH2*	c.942 + 2dupT		N	L	L	N	No	F	AC	LS	II
1.18	*MSH2*	c.942 + 6A > T		N	L	L	N	Yes	F	AC	RS	I
1.19	*MSH6*	c.3261dupC	p.(Phe1088Leufs*5)	N	N	L	N	Yes	M	AC	RS	III
1.20	*PMS2*	c.613C > T	p.(Gln205Ter)	N	N	N	L	No	F	AC	RS	II
1.21	*MUTYH*	c.1214C > T	p.(Ala405Val)	N	N	N	N	NA	F	AC	LS	III
1.22	*MUTYH*	c.536A > G	p.(Tyr179Cys)	N	N	N	N	Yes	F	AC	LS	IV
Multicarrier												
1.23	*APC* *MUTYH* *MUTYH*	c.1748C > A c.536A > G c.1187G > A	p.(Ser583Ter) p.(Tyr179Cys) p.(Gly396Asp)	N	N	N	N	No	F	AC	LS	III
1.24	*MLH1* *MUTYH*	c.1667 + 2_1667 + 8delinsATTT c.1187G > A	p.(Gly396Asp)	L	N	N	L	Yes	M	MUC	RS	I

Abbreviations: FDR = First degree relative; HIS = Histology; TL = Tumor localization; UICC = Union for International Cancer Control; L = Loss of expression; N = Normal expression; NA = Not available; F = Female; M = Male; AC = Adenocarcinoma; SRC = Signet ring cell carcinoma; MUC = Mucinous adenocarcinoma; LS = Left-sided, RS = Right-sided, SY = Synchronous; RE = rectum. The Locus Reference Genomic (LRG) records used for reporting sequence variants with clinical implications: *MLH1* (LRG_216), *MSH2* (LRG_218), *PMS2* (LRG_161), *APC* (LRG_130t1), *MUTYH* (LRG_220), *MSH6* (LRG_219).

**Table 2:** Six patients with no pathogenic germline variant but with deficient mismatch repair.

Id	MLH1	MSH2	MSH6	PMS2	HYP	BRAF	Gene	Variant (c.)	Variant (*p*.)	FDR	Sex	HIS	TL	UICC
2.1	L	N	N	L	NEG	NA	MLH1	c.1996T > C	p.(Tyr721His)	NA	F	AC	RS	II
2.2	N	N	N	L	NR	NR	RNF43	c.989G > A	p.(Arg330Gln)	NA	M	MUC	LS	IV
2.3	N	L	L	N	NR	NR	MSH3	c.2732T > G	p.(Leu911Trp)	No	F	MUC	LS	I
2.4	L	N	N	L	NA	NEG	PMS2	c.857A > G	p.(Asp286Gly)	No	M	MED	LS	II
2.5	L	N	N	L	NA	NA				NA	M	AC	LS	III
2.6	L	N	NA	NA	NA	NA	MSH2	c.1275A > G	p.(Glu425=)	No	F	SRC	SY	IV

Abbreviations: HYP = Hypermethylation of MLH1; BRAF = BRAF mutation; VUS = Variant of uncertain significance; FDR = First degree relative; HIS = Histology; TL = Tumor localization; UICC = Union for International Cancer Control; L = Loss of expression; N = Normal expression; NEG = Negative; NA = Not available; NR = Not relevant; F = Female; M = Male; AC = Adenocarcinoma; SRC = Signet ring cell carcinoma; MUC = Mucinous adenocarcinoma; MED = Medullary adenocarcinoma; RS = Right-sided; LS = Left-sided; SY = Synchronous. The Locus Reference Genomic (LRG) records used for reporting sequence variants with clinical implications: *MLH1* (LRG_216), *RNF43* (LRG_1026), *MSH3* (RefSeq NM_002439.5), *PMS2* (LRG_161), *MSH2* (LRG_218)

**Table 3:** Comparision between patients and tumor characteristics in young colorectal cancer patients with pathogenic germline variants (PGV) and no pathogenic variant (NPGV). Significant differences are highlighted with bold text.

Patient Characteristics	PGV *n* = 24	NPGV *n* = 74	*p*	All Patients *n* = 98
**Sex**			0.802	
Female	14 (58.3)	41 (55.4)		55 (56.1)
Male	10 (41.7)	33 (44.6)		43 (43.9)
**Location**			**0.001**	
Right-sided	14 (58.3)	22 (29.7)		36 (36.7)
Left-sided	6 (25.0)	25 (33.8)		31 (31.6)
Rectum	1 (4.2)	26 (35.1)		27 (27.5)
Synchronous	3 (12.5)	1 (1.4)		4 (4.1)
**Histology**			0.708	
Adenocarcinoma	21 (87.5)	56 (75.7)		77 (78.6)
Mucinous	2 (8.3)	11 (14.9)		13 (13.3)
Signet-ring cell	1 (4.2)	6 (8.1)		7 (7.1)
Medullary carcinoma	0 (0.0)	1 (1.3)		1 (1.0)
**Tumor grade (only adenocarcinomas)**			0.056	
Moderately differentiated	13 (61.9)	48 (85.7)		61 (79.2)
Poorly differentiated	5 (23.8)	6 (10.7)		11 (14.3)
Not assessed	3 (14.3)	2 (3.6)		5 (6.5)
**UICC**			**0.016**	
I	6 (25.0)	14 (18.9)		20 (20.4)
II	11 (45.8)	14 (18.9)		25 (25.5)
III	6 (25.0)	27 (36.5)		33 (33.7)
IV	1 (4.2)	19 (25.7)		20 (20.4)
**MMR**			**<0.0001**	
pMMR	4 (16.7)	68 (91.9)		72 (73.5)
dMMR	20 (83.3)	6 (8.1)		26 (26.5)
**First degree relative with CRC**			**0.004**	
Yes	13 (54.2)	16 (21.6)		29 (29.6)
No	6 (25.0)	45 (60.8)		51 (52.0)
Unknown	5 (20.8)	13 (17.6)		18 (18.4)
**Tumor–stroma ratio**			0.092	
High	19 (79.2)	44 (59.5)		63 (64.3)
Low	5 (20.8)	30 (40.5)		35 (35.7)
**Venous invasion**			0.112	
Yes	3 (12.5)	22 (29.7)		73 (74.5)
No	21 (87.5)	52 (70.3)		25 (25.5)
**Perineural invasion**			0.347	
Yes	2 (8.3)	13 (17.6)		15 (15.3)
No	22 (91.7)	61 (82.4)		83 (84.7)
**Tumor budding**			0.172	
Low	15 (62.5)	39 (52.7)		54 (55.1)
Intermediate	6 (25.0)	12 (16.2)		18 (18.4)
High	3 (12.5)	23 (31.1)		26 (26.5)
**T-stage**			0.121	
T1	4 (16.7)	3 (4.0)		7 (7.1)
T2	3 (12.5)	12 (16.2)		15 (15.3)
T3	14 (58.3)	39 (52.7)		53 (54.1)
T4	3 (12.5)	20 (27.0)		23 (23.5)
**N-stage**			**0.007**	
N0	17 (70.8)	28 (37.8)		45 (45.9)
N1	4 (16.7)	13 (17.6)		17 (17.4)
N2	3 (12.5)	33 (44.6)		36 (36.7)
**Surgical resection**			**<0.0001**	
Extended surgery	11 (45.8)	6 (8.1)		17 (17.4)
Segmental resection	13 (54.2)	68 (91.9)		81 (82.6)

PGV = Patients with pathogenic germline variant; NPGV = Patients with no pathogenic variant; UICC = Union for International Cancer Control; MMR = Mismatch repair; CRC = Colorectal cancer; pMMR = proficient MMR; dMMR = deficient MMR.

## Data Availability

The data presented in this study are available on request from the corresponding author. The data are not publicly available due to privacy and ethical reasons.
